# Incidental Diagnosis of a Gossypiboma With Complete Transmural Migration Into the Jejunum After a Hysterectomy: A Case Report

**DOI:** 10.7759/cureus.114054

**Published:** 2026-08-06

**Authors:** Edgar Alexis Flores García, José L Busquets Gallegos, Francisco J Rivera Vazquez, Hector X Rodríguez Hernández, Oscar E Coronado Padilla, Jenniffer Luján Hurtado, Abigail Diaz Alba

**Affiliations:** 1 Surgery, Hospital General Nuevo Gómez Palacio, Gómez Palacio, MEX

**Keywords:** gossypiboma, small bowel mass, small bowel obstruction (sbo), small bowel resection, transmural migration

## Abstract

Gossypiboma, or retained surgical sponge, is a rare and preventable surgical complication. Complete transmural migration of a retained sponge into the gastrointestinal tract without overt intestinal perforation is an exceptionally unusual phenomenon that poses significant diagnostic and therapeutic challenges. A 41-year-old woman with a history of open hysterectomy seven months prior presented with progressive neurological deterioration, severe metabolic encephalopathy, and suspected urosepsis, without classical signs of mechanical bowel obstruction. Abdominal computed tomography (CT) demonstrated a pelvic intestinal loop with marked wall thickening and a radiopaque structure preoperatively misidentified as a metallic foreign body within the sigmoid colon. Exploratory laparotomy revealed a dense inflammatory mass involving a matted jejunal loop 60 cm distal to the ligament of Treitz. A 50 cm non-viable jejunal segment was resected, and a double-barrel jejunostomy was performed. Longitudinal opening of the resected specimen revealed a retained surgical sponge completely within the jejunal lumen with spontaneous sealing of the bowel wall. Despite surgical intervention and intensive care support, the patient succumbed to refractory septic shock and multi-organ dysfunction on the first postoperative day. This case illustrates an atypical presentation of a gossypiboma manifesting as chronic systemic inflammation, severe hypoalbuminemia, and metabolic encephalopathy rather than classical bowel obstruction. Documenting this case highlights the diagnostic pitfalls of CT interpretation, where radiopaque markers can mimic metallic foreign bodies or cause anatomical misattribution due to pelvic adhesions, and emphasizes the vital necessity of maintaining a high index of suspicion in any patient with prior abdominal surgery and unexplained systemic decline.

## Introduction

Gossypiboma, also termed textiloma or retained surgical sponge, is a rare yet potentially severe complication of surgical procedures. It denotes the inadvertent retention of surgical textiles, predominantly sponges or gauze, within a body cavity. Although considered a preventable “never event,” its occurrence persists despite established intraoperative protocols such as manual sponge counting, surgical safety checklists, and emerging tracking technologies. The clinical significance of gossypiboma stems from its highly variable presentation, which ranges from incidental asymptomatic findings to life-threatening complications, including intra-abdominal abscesses, bowel perforation, mechanical obstruction, and systemic sepsis [1,2].

The clinical course of gossypiboma is largely influenced by the type of foreign body reaction, which may be exudative, leading to early symptomatic presentation, or aseptic fibrotic, resulting in delayed and often silent evolution. In chronic cases, the inflammatory response may lead to progressive adhesion formation and erosion into adjacent structures, particularly the gastrointestinal tract. One of the most unusual and clinically significant manifestations is transmural migration into the intestinal lumen, a rare process in which the retained sponge gradually penetrates the bowel wall and becomes entirely intraluminal [1,3].

Transmural migration is thought to occur secondary to persistent foreign body reaction, pressure necrosis, and chronic inflammation, ultimately allowing the sponge to erode through the intestinal wall. Once intraluminal, the foreign body may act as a lead point for obstruction, or remain partially mobile, producing intermittent or subacute obstructive symptoms. In some cases, the bowel wall defect may seal spontaneously, leaving no obvious perforation at the time of surgical exploration, which can make intraoperative diagnosis challenging [3].

Preoperatively identifying a gossypiboma remains notoriously difficult. Its clinical features are often nonspecific and frequently overlap with more routine postoperative issues, such as intra-abdominal abscesses or urinary tract infections. Computed tomography (CT) serves as the diagnostic modality of choice; however, interpreting CT findings can be complex. Radiopaque markers within surgical sponges can easily mimic metallic foreign bodies, surgical clips, or dystrophic calcifications [4]. Consequently, maintaining a high index of suspicion in any patient with a history of prior abdominal surgery is vital for accurate radiological interpretation and timely intervention.

Given its rarity and wide spectrum of presentation, gossypiboma continues to represent a diagnostic and therapeutic challenge in surgical practice. The present case is reported due to the unusual presentation of a retained surgical sponge with complete transmural migration into the jejunum, incidentally identified during the evaluation of a patient without classic signs of mechanical bowel obstruction. This case highlights both the diagnostic difficulty and the importance of maintaining a high index of suspicion in patients with previous abdominal surgery, even when presenting with atypical clinical manifestations.

## Case presentation

A 41-year-old woman was brought to the emergency department in July 2026 with a seven-day history of progressive neurological deterioration characterized by loss of speech, asthenia, and generalized weakness. According to her family, she had experienced progressive gait impairment and somnolence over the preceding six months. One month before admission (May 2026), she sustained a fall associated with transient loss of consciousness and retrograde amnesia, without receiving medical attention.

Her past medical history was unremarkable. She had no known chronic diseases or drug allergies. Surgical history included two previous cesarean sections performed 20 and 15 years earlier, and an open hysterectomy with bilateral salpingo-oophorectomy performed at another medical facility approximately seven months before (December 2025) admission for an unspecified indication.

On physical examination, the patient was confused and able to follow only simple commands. She presented with generalized jaundice, obesity with a prominent abdominal pannus, bilateral upper and lower extremity edema, and multiple ecchymoses. She was afebrile. The abdomen was soft and non-distended, without signs of peritoneal irritation, with tenderness on deep palpation in the right upper quadrant.

Initial laboratory evaluation demonstrated abnormalities consistent with a systemic inflammatory response and significant hepatic dysfunction. Hematologic analysis revealed leukocytosis with neutrophilia, accompanied by mild anemia and a normal platelet count. Biochemical testing showed elevated blood urea nitrogen and urea levels, while coagulation studies demonstrated prolonged prothrombin time, an elevated international normalized ratio, and mildly prolonged activated partial thromboplastin time, suggesting impaired synthetic liver function. Liver function tests were notable for marked hyperbilirubinemia, predominantly direct, together with severe hypoalbuminemia and reduced total protein levels. In addition, the lipid profile revealed severe hypertriglyceridemia with low high-density lipoprotein cholesterol (Table 1).

**Table 1:** Initial laboratory findings on hospital admission. The table summarizes the patient’s hematologic, biochemical, coagulation, liver function, and lipid profile results obtained during the initial evaluation. Abnormal values reflect systemic inflammation, hepatic dysfunction with impaired synthetic function, hyperbilirubinemia, severe hypoalbuminemia, and hypertriglyceridemia.

Parameter	Patient value	Normal reference range (adults)
Hematology		
White blood cells	13.33 × 10³/µL	4.5–11.0 × 10³/µL
Neutrophils	71.88%	40–70%
Hemoglobin	10.03 g/dL	12.0–15.5 g/dL (female); 13.5–17.5 g/dL (male)
Hematocrit	28.67%	37–48% (female) 45 – 52% (male)
Platelets	356 × 10³/µL	150–450 × 10³/µL
Routine chemistry		
Glucose	93 mg/dL	70–100 mg/dL
Blood urea nitrogen	66 mg/dL	7–20 mg/dL
Urea	141.2 mg/dL	15–45 mg/dL
Coagulation panel		
Prothrombin time	27.5 seconds	11.0–13.5 seconds
Activated partial thromboplastin time	30.7 seconds	25.0–35.0 seconds
International normalized ratio	2.35	0.8–1.1 (without anticoagulation)
Fibrinogen	218 mg/dL	200–400 mg/dL
Liver function tests		
Total bilirubin	6.3 mg/dL	0.2–1.2 mg/dL
Direct bilirubin	3.3 mg/dL	0.0–0.3 mg/dL
Indirect bilirubin	0.8 mg/dL	0.2–0.8 mg/dL
Total protein	3.5 g/dL	6.0–8.3 g/dL
Albumin	2.1 g/dL	3.5–5.0 g/dL
Lipid profile		
Total cholesterol	201 mg/dL	<200 mg/dL
Triglycerides	672 mg/dL	<150 mg/dL
High-density lipoprotein cholesterol	36 mg/dL	>40 mg/dL (female); >50 mg/dL (male)
Low-density lipoprotein cholesterol	31 mg/dL	<100 mg/dL
Very-low-density lipoprotein cholesterol	134 mg/dL	2–30 mg/dL

Specific acute-phase inflammatory biomarkers, such as C-reactive protein and procalcitonin, were not obtained during the initial emergency evaluation due to the expedited surgical decision-making process.

**Figure 1:**
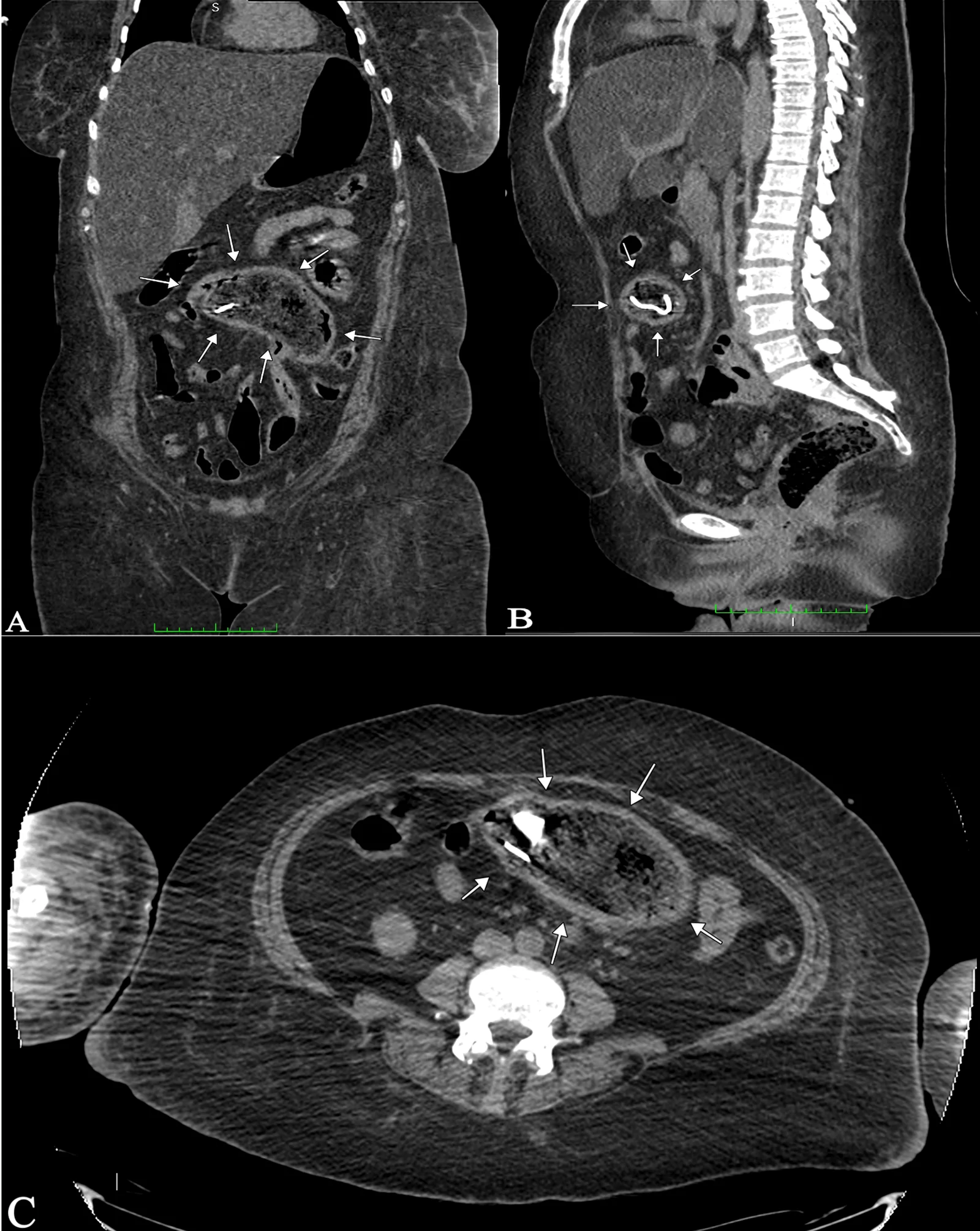
Abdominal and pelvic computed tomography (CT) scan in soft-tissue windows. CT images demonstrating the retained intraluminal foreign body within an inflamed intestinal loop in the lower abdomen/pelvis, preoperatively misidentified as the sigmoid colon due to severe pelvic adhesions and mass effect. White arrows delineate the affected loop, characterized by marked wall thickening and surrounding perilesional fat stranding. (A) Coronal reconstruction: Coronal CT reconstruction showing focal distension of the pelvic intestinal loop and inflammatory involvement of adjacent fat (white arrows). The hyperdense radiopaque marker of the sponge mimics an elongated metallic object within the lumen. (B) Sagittal reconstruction: Sagittal CT reconstruction illustrating the intrinsic curvature of the foreign body and its close contact with the bowel mucosa (white arrows). (C) Axial view: Axial CT image at the level of the lesser pelvis confirming concentric wall thickening and marked fat stranding (white arrows) secondary to severe local inflammation.

Urology consultation requested CT urography, which demonstrated bilateral nephrolithiasis, significant fecal loading in the sigmoid colon, and an irregular, flattened radiopaque structure surrounded by marked wall thickening and inflammatory fat stranding within the pelvis. Due to severe pelvic adhesions from previous gynecological surgery and localized mass effect, this deep pelvic loop was preoperatively misidentified on CT as the sigmoid colon containing a metallic foreign body (Figure 1). No free intraperitoneal or pelvic fluid was identified.

This chronic, seven-month indolent foreign-body reaction and progressive transmural migration induced a low-grade, sustained systemic inflammatory response syndrome. The resulting severe hypoalbuminemia, coupled with sepsis-induced hepatic dysfunction (marked direct hyperbilirubinemia and coagulopathy), led to metabolic and septic encephalopathy. This pathophysiological cascade directly accounted for the patient’s six-month history of insidious physical decline, progressive gait impairment, and acute altered mental status upon admission

General Surgery was consulted due to the presence of an unexplained intra-abdominal foreign body in a patient with prior abdominal surgery. Although the patient did not exhibit classic signs of mechanical bowel obstruction, exploratory laparotomy was performed to identify and remove the foreign body.

Intraoperatively, a dense inflammatory mass with multiple adhesions between jejunal loops approximately 60 cm distal to the ligament of Treitz was identified. Careful adhesiolysis revealed a jejunal segment with ischemic and necrotic changes compromising bowel viability. Approximately 50 cm of jejunum was resected. Given the patient’s critical condition, severe hypoalbuminemia, coagulopathy, and sepsis, primary anastomosis was avoided, and a double-barrel jejunostomy was performed due to high risk of anastomotic leakage.

**Figure 2:**
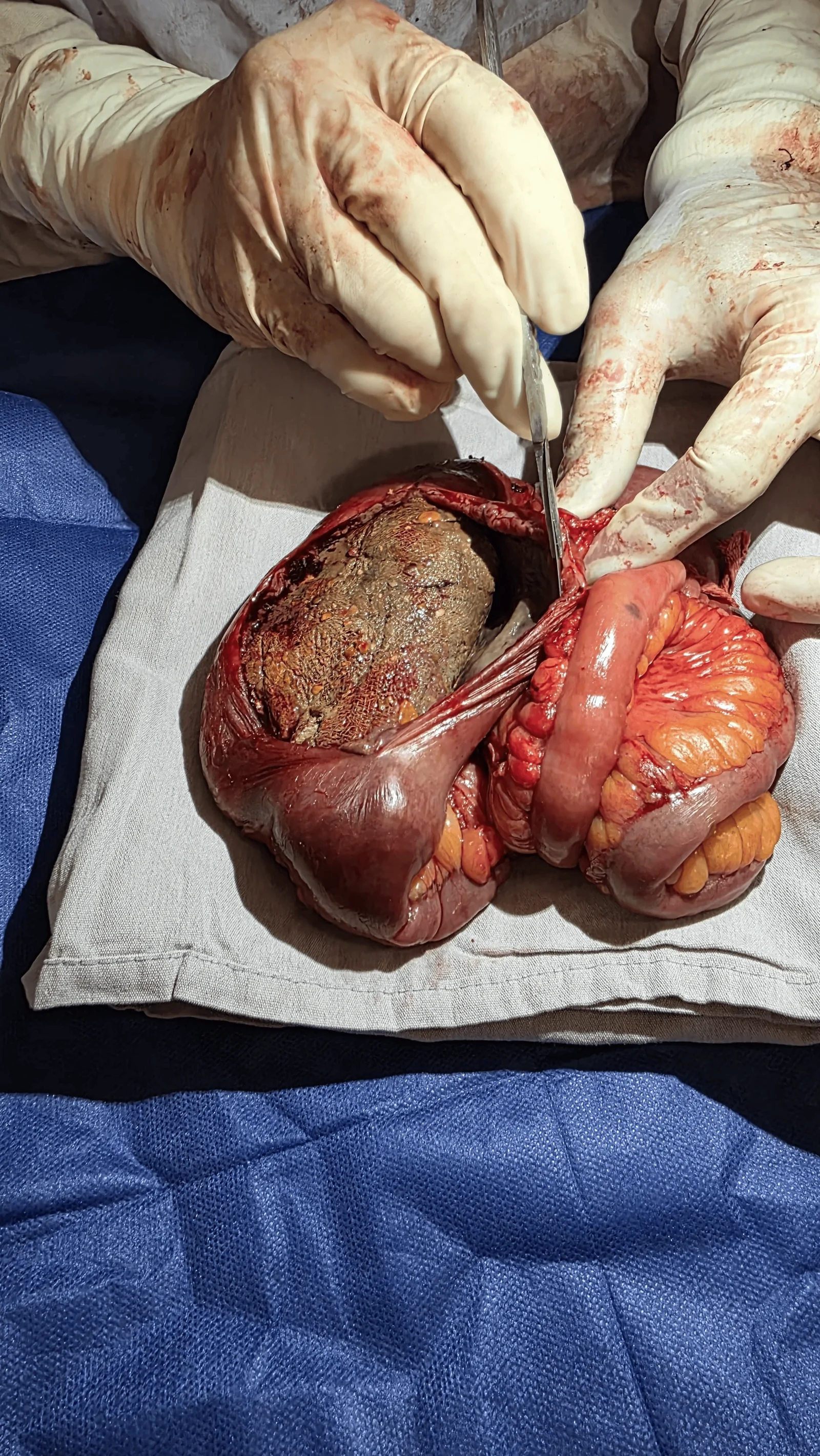
Surgical macro-photograph of a 50 cm jejunal resection specimen. A longitudinal incision (indicated by the scalpel in the right surgical hand) reveals a perfectly formed intraluminal gossypiboma. The foreign body exhibits an ovoid morphology and a textile texture with a brownish-green discoloration, indicative of impregnation by enteric contents. The surrounding intestinal wall shows marked inflammatory thickening.

On opening the resected specimen, a surgical sponge was found completely within the jejunal lumen, with no evidence of active perforation or communication with the peritoneal cavity, consistent with complete transmural migration of a retained surgical sponge (Figure 2). The radiopaque marker corresponded to the previously described “metallic” density seen on CT, which likely represented intermittent pseudo-obstruction before complete intestinal occlusion.

Following completion of the double-barrel jejunostomy, the patient was transferred to the Intensive Care Unit requiring high-dose vasopressor support and mechanical ventilation. Postoperative laboratory monitoring demonstrated refractory metabolic deterioration, characterized by worsening lactic acidosis, severe uncorrectable metabolic acidosis, and progressive coagulopathy. Despite aggressive supportive care, the patient suffered refractory septic shock complicated by multi-organ dysfunction syndrome, leading to fatal cardiac arrest on the first postoperative day (total postoperative follow-up: 24 hours). An autopsy was declined by the patient’s family.

This case illustrates a critical diagnostic caveat: in patients with a history of abdominal surgery, gossypiboma and transmural migration should remain in the differential diagnosis when presenting with unexplained chronic systemic inflammation, severe hypoalbuminemia, or metabolic encephalopathy, even in the complete absence of peritoneal signs or classic mechanical bowel obstruction.

## Discussion

Gossypiboma remains an uncommon but clinically significant complication of abdominal surgery, with variable clinical presentations depending on the host inflammatory response and the anatomical site of migration. In the present case, the patient demonstrated an atypical course with systemic deterioration and neurological compromise rather than classic abdominal manifestations, which contributed to a delayed recognition of the underlying condition [5].

The clinical course in this patient illustrates how a retained surgical sponge can remain localized for months while causing profound systemic consequences. The six-month period of silent transmural erosion into the jejunum initiated a continuous inflammatory cascade and localized ischemic injury. Rather than presenting with classic signs of acute mechanical bowel obstruction, the persistent foreign-body reaction and bacterial translocation generated chronic systemic inflammation, progressive malnutrition, and liver dysfunction [6]. Ultimately, this chronic inflammatory state culminated in severe hypoalbuminemia, coagulopathy, and septic/metabolic encephalopathy, providing a clear pathophysiological link between the silent intra-abdominal process and the patient’s severe physical decline and altered mental status.

Transmural migration of retained surgical sponges into the gastrointestinal tract is a rare but well-described phenomenon. It is thought to result from chronic foreign-body reaction, progressive adhesion formation, pressure necrosis, and gradual erosion through the bowel wall until the sponge becomes completely intraluminal. In some cases, the defect in the bowel wall may seal spontaneously, leaving no evident perforation at surgical exploration, as observed in this case [7].

Clinically, transmural migration may present with nonspecific abdominal symptoms or with acute complications such as intestinal obstruction or peritonitis. Agrawal et al. described a case in which transmural migration resulted in an acute abdomen requiring emergency surgical intervention [8]. Adôrno et al. reported imaging findings that may suggest the diagnosis preoperatively, although radiologic interpretation can be challenging depending on the characteristics of the retained material [9]. Baset et al. highlighted cases in which intraluminal migration led to acute bowel obstruction with sudden clinical deterioration, emphasizing the potential for abrupt and severe presentations [10].

CT remains the imaging modality of choice for suspected gossypiboma; however, findings are often nonspecific and may be misinterpreted, particularly when radiopaque markers mimic other foreign bodies. Obeidat et al. noted that intraluminal gossypibomas are frequently diagnosed intraoperatively due to their variable and often misleading radiologic appearance [11].

Management depends on the clinical condition of the patient, the location of the foreign body, and the presence of complications. Surgical resection is generally required in cases complicated by bowel compromise. In the present case, segmental jejunal resection with diversion was performed due to severe sepsis, hypoalbuminemia, and coagulopathy, which increased the risk of primary anastomotic failure [12].

Overall, gossypiboma remains a preventable but potentially severe complication. Early recognition requires a high index of suspicion, particularly in patients with a history of prior abdominal surgery and atypical clinical or radiologic findings. This case reinforces the importance of considering retained surgical foreign bodies in the differential diagnosis of nonspecific abdominal or systemic deterioration.

From a medicolegal and patient safety perspective, gossypiboma represents a highly preventable “never event.” Mitigating this risk requires strict adherence to standardized intraoperative sponge counting protocols alongside routine visual inspection, supplemented by technological advances such as intraoperative radiopaque marker detection via plain radiographs, barcode tracking, or radiofrequency tagging before surgical closure [13].

## Conclusions

Complete transmural migration of a retained surgical sponge into the jejunum can remain clinically silent for months, presenting atypically as chronic systemic inflammation, severe hypoalbuminemia, and metabolic encephalopathy rather than classical mechanical bowel obstruction. This case highlights two critical take-home lessons: first, CT radiopaque markers within surgical sponges can easily mimic metallic foreign bodies and cause anatomical misattribution due to severe pelvic adhesions; second, maintaining a high index of suspicion in any patient with prior abdominal surgery and unexplained systemic decline is essential to enable prompt surgical exploration before irreversible septic shock and multi-organ dysfunction occur.
